# Supplementary material for: Comprehensive feature evaluation of the main facades of Catholic churches in Sichuan-Chongqing region based on semantic difference method, 1840–1949
Source: PLoS One. 2026 May 8;21(5):e0348806. doi: 10.1371/journal.pone.0348806 (PMC13155555; doi:10.1371/journal.pone.0348806)
Supplement: S1 Appendix — (DOCX) [file pone.0348806.s001.docx]

**Appendix**

$\alpha=\frac{K}{K-1}\times\left(1-\frac{\sum_{i=1}^{K} \sigma_{i}^{2}}{\sigma_{T}^{2}}\right)$ (Formula 1)

In Cronbach's Alpha coefficient formula, K denotes the number of items in the scale, $\sigma_{i}^{2}$represents the variance of the i-th item's score, and $\sigma_{T}^{2}$ represents the variance of the total scores of all items.

$KMO=\frac{\sum_{i\neq j} r_{ij}^{2}}{\sum_{i\neq j} r_{ij}^{2}+\sum_{i\neq j} \alpha_{ij}^{2}}$ (Formula 2)

In the KMO calculation formulas, $r_{ij}^{2}$represents the square of the correlation coefficient between item i and item j, while$\alpha_{ij}^{2}$ represents the square of their partial correlation coefficient.

$F_{n}(x)=\frac{1}{n}\sum_{i=1}^{n} I(X_{i}\text{≤x})$ (Formula 3)

$D\text{n}=\sup|F_{\text{n}}\text{(x)}-\text{F(x)}|$ (Formula 4)

In the *Dn* calculation formula, Dn denotes the K-S statistic, *Fn(X)* represents the empirical distribution function of the sample, *F(x)*is the cumulative distribution function of the theoretical distribution, I is the indicator function (taking 1 if *X_i_*≤*x*, 0 otherwise), and *n* is the sample size.

$p=2\sum_{\text{k}=1}^{\infty} (-1)^{k-1}\exp(-2k^{2}D\text{n}^{2}n)$ (Formula 5)

where $p$ represents significance.

$\rho=1-\frac{6\sum\text{d}_{\text{i}}^{2}}{\text{n（n}^{2}\text{-1）}}$ (Formula 6)

ρ In the calculation formula, ρ represents the Spearman's correlation coefficient, *d_i_* denotes the difference in rank between the *i*-th data point and the two variables, and *n* is the total sample size.
